# Acquired dysgraphia in adults following right or left-hemisphere stroke

**DOI:** 10.1590/S1980-57642014DN83000007

**Published:** 2014

**Authors:** Jaqueline de Carvalho Rodrigues, Denise Ren da Fontoura, Jerusa Fumagalli de Salles

**Affiliations:** 1 Psychologist, Master Degree and Doctoral Student in Psychology, Graduate Department of Psychology, Federal University of Rio Grande do Sul – UFRGS, RS, Brazil.; 2 Speech/Language Pathologist, PhD in Psycholinguistics, Master Degree in Neuroscience, Specialist in Speech Pathology Rehabilitation, Post graduated in Neuropsychology, RS, Brazil.; 3 Speech/Language Pathologist, Master Degree and PhD (Psychology), Adjunct Professor of the Department of Developmental and Personality Psychology, Graduate Department of Psychology, Federal University of Rio Grande do Sul - UFRGS, Head of the Cognitive Neuropsychology Research Group - NEUROCOG., RS, Brazil

**Keywords:** agraphia, cognitive neuropsychology, written language, cerebral dominance

## Abstract

**Objective:**

This study aimed to assess the strengths and difficulties in word and pseudoword writing in adults with left- and right-hemisphere strokes, and discuss the profiles of acquired dysgraphia in these individuals.

**Methods:**

The profiles of six adults with acquired dysgraphia in left- or right-hemisphere strokes were investigated by comparing their performance on word and pseudoword writing tasks against that of neurologically healthy adults. A case series analysis was performed on the patients whose impairments on the task were indicative of acquired dysgraphia.

**Results:**

Two patients were diagnosed with lexical dysgraphia (one with left hemisphere damage, and the other with right hemisphere damage), one with phonological dysgraphia, another patient with peripheral dysgraphia, one patient with mixed dysgraphia and the last with dysgraphia due to damage to the graphemic buffer. The latter patients all had left-hemisphere damage (LHD). The patterns of impairment observed in each patient were discussed based on the dual-route model of writing.

**Conclusion:**

The fact that most patients had LHD rather than right-hemisphere damage (RHD) highlights the importance of the former structure for word processing. However, the fact that lexical dysgraphia was also diagnosed in a patient with RHD suggests that these individuals may develop writing impairments due to damage to the lexical route, leading to heavier reliance on phonological processing. Our results are of significant importance to the planning of writing interventions in neuropsychology.

## Abstract (pt)

**Objetivo:**

Investigar aspectos preservados e dificuldades na escrita de palavras e pseudopalavras em adultos que sofreram acidente vascular cerebral (AVC) à esquerda e à direita e discutir os perfis de disgrafia adquirida nesses indivíduos.

**Métodos:**

Investigaram-se perfis de disgrafia adquirida a partir da avaliação das habilidades e dificuldades na escrita de palavras e pseudopalavras de seis adultos que sofreram AVC no hemisfério direito (LHD) e no hemisfério esquerdo (LHE), comparados a adultos neurologicamente saudáveis. Realizou-se análise de séries de casos com os pacientes que apresentaram desempenho deficitário na escrita de palavras, que indicavam a presença de uma disgrafia adquirida.

**Resultados:**

Foram identificados dois casos com disgrafia lexical (sendo um com LHE e outro com LHD), um caso com disgrafia fonológica, um com disgrafia periférica, um com disgrafia mista e um com disgrafia por déficit no *buffer* grafêmico, todos estes com LHE. Destacou-se nesse estudo a heterogeneidade das habilidades linguísticas dos casos clínicos, discutidas de acordo com o modelo cognitivo de dupla-rota de escrita.

**Conclusão:**

O maior prejuízo encontrado nos pacientes com LHE ressalta a importância desse hemisfério cerebral para o processamento da escrita de palavras. A presença de um caso com LHD com perfil de disgrafia lexical destaca a necessidade de melhor estudar o papel do hemisfério direito no processamento de palavras. Espera-se que esse estudo contribua para o planejamento de estratégias de intervenção neuropsicológica direcionadas à escrita de palavras.

## INTRODUCTION

Acquired dysgraphia (or agraphia) is the partial or total inability to produce written language following neurological damage.^1,2^ According to cognitive models of writing, dysgraphia may be either a result of language impairment^3-5^ or of praxis and visuospatial dysfunction.^7-9^ According to the dual-route or multiple route model, one of the most widespread and well-accepted models of writing,^10-14^ written word production may occur either through phonological mediation (the conversion of phonemes to graphemes) or by direct lexical access. While the phonological route is involved mainly in the production of unfamiliar words and pseudowords, the lexical route is preferred when writing familiar words, and the only suitable means of writing irregular words.^15^

The different types of dysgraphia caused by distinct patterns of impairment in one or more components of the dual route model can be identified by the assessment of psycholinguistic effects in writing (word length, regularity, frequency, etc.) and of the types of errors observed in word/pseudoword writing.^15^ According to a cognitive model of writing to dictation in the Brazilian Portuguese language developed by Lecours and Parente,^16^ dysgraphia can be divided into the following subtypes: central (lexical, phonological, semantic and deep dysgraphia) and peripheral (in the case of damage to the graphemic or allographic buffers, or impairments in the planning and execution of hands articulatory movements).^6^

Lexical dysgraphia occurs when there is damage to the lexical route and heavy reliance on phonological writing strategies, leading to adequate performance in tasks involving words and pseudowords, but difficulty writing ambiguous and irregular words. Regularization errors are common in these patients, as is the tendency to perform better when writing frequent as opposed to infrequent words.^15^ This type of dysgraphia is often caused by lesions to the left parietal lobe.^4^

Phonological dysgraphia is associated with extreme difficulty in writing pseudowords as compared to real words due to impaired phoneme-grapheme conversion, which may also lead to problems when writing unfamiliar words.^17^ As a result, lexicalization errors, as well as frequency and lexicality effects, are often observed in these cases. This type of dysgraphia is often reported in patients with damage to perisylvian cortical regions.^18,19^

Mixed (or global) dysgraphia is associated with impairment to both lexical and phonological mechanisms. Patients with this condition are able to write some regular words but have difficulty writing irregular words and pseudowords.^20,21^ Semantic dysgraphia refers to the inability to attribute meaning to written words, and is often observed following left hemisphere lesions.^5^ Deep dysgraphia is associated with phonological deficits, which lead to semantic paragraphias, lexicality effects, and difficulty writing pseudoword, as well as unfamiliar and abstract words.^22^ Semantic dysgraphia has been found to occur following extensive lesions to the supramarginal gyrus and insula.^1^

Peripheral dysgraphia caused by damage to the graphemic buffer leads to difficulties in lexical access and phoneme-grapheme conversion during the writing process.^23,24^ Patients with this condition retain the ability to write well-formed graphemes, although the substitution, omission, addition or transposition of letters within words may be observed.^24^ Damage to the allographic buffer, on the other hand, tends to impair the grapheme selection process, which often results in the use of both upper and lower case letters and cursive and block letters in the same word.^26^ These difficulties may be associated with damage to the left temporo-parietooccipital cortex.^2^ Lastly, apraxic dysgraphia is caused by alterations in the planning and generation of the motor sequences required to write letters.^6^ This condition is believed to be caused by damage to the left parietal cortex.^28^

Language impairments following left hemisphere damage (LHD) have been extensively investigated in the literature. However, few studies have investigated the performance of patients with right hemisphere damage (RHD) on word writing tasks. Furthermore, the qualitative nature of language impairment in dysgraphia has only been scarcely studied.^24^ Therefore, this study aimed to assess the strengths and difficulties in word and pseudoword writing in adults with left- and right-hemisphere strokes, and discuss the profiles of acquired dysgraphia in these individuals.

## METHOD

**Participants.** This was a series of case studies^39^ involving six patients, Brazilian-Portuguese native speakers, with acquired dysgraphia following stroke. Five of the patients had LHD while one had RHD. These patients were drawn from a sample of 40 right-handed adults who completed writing tasks, ten of whom had LHD (M=59.2; SD=8.6 years old), ten had RHD (M=53.3; SD=9.7 years old) and 20 were neurologically healthy (M=55.7; SD=9.3 years old). Control participants were matched to patients by gender, age and years of education. Dysgraphia was considered when patients obtained a score below two standard-deviations from the control mean in a word/pseudoword writing task or when the number of errors on the task was over two standard-deviations above the control mean (Z score). The cases selected had distinct sociodemographic characteristics, which are displayed in Table 1.

**Table 1:** Patient sociodemographic data.

Case	Gender	Age (years)	Years of education	RW Habits*	Occupation	Socioeconomic Status**
LHD1	F	58	5	Low	Housewife	C1
LHD2	F	73	4	High	Housewife	C2
LHD3	F	48	9	Low	Secretary	C1
LHD4	M	67	8	Low	Doorman	C2
LHD5	M	50	11	Low	Taxi Driver	C1
RHD6	F	61	4	Low	Housekeeper	C1

LHD: left hemisphere damage; RHD: right hemisphere damage; M: male; F: female; R: reading; W=writing.

* Scores between 0 and 13 were indicative of a low frequency of reading and writing, while scores between 14 and 28 corresponded to frequent reading and writing habits. This variable was assessed by a reading and writing inventory, published by Pawlowski et al., 2012.

** Assessed according to the Brazilian Economic Classification Criteria (ABEP, 2012).

The type and location of lesions observed in each patient are described in Table 2. Two of the patients with LHD had Broca's Aphasia (LHD3 and LHD4), while one patient had transcortical motor aphasia (LHD5).

**Table 2:** Patient neurological data.

Case	Etiology	Region of stroke	Location of stroke	Months since stroke
LHD1	H	Subcortical	Basal Ganglia	28
LHD2	I	Subcortical	Parieto-occipital	24
LHD3	I	Cortico subcortical	Fronto-temporal	70
LHD4	I	Cortico subcortical	Fronto-temporal	18
LHD5	H	Subcortical	Insula and periventricular region	48
RHD6	H	Cortical	Frontal	22

LHD: left hemisphere damage; RHD: right hemisphere damage; I: ischemic; H: hemorrhagic.

**Instruments and procedures.** All participants or caregivers provided written informed consent prior to enrollment in the study, which was approved by the local ethics committee. The patients did not have severe depression (Yesavage Geriatric Depression Scale – GDS-15^32^ or Beck Depression Inventory BDI-II)^33^ or impairments in language comprehension (Token Test – short version).^35^ Furthermore, patients were not aphasic and had predominantly expressive language impairment (Boston Aphasia Diagnostic Test - short version)^29,30^

Patients were administered the spoken and written language subtests of the Brazilian Brief Neuropsycholinguistic Assessment Battery for Expressive Aphasia (NEUPSILIN-Af).^36,37^ The spoken language subtests included in this battery assess Automatic Language, Naming, Repetition, Spoken Comprehension and Inferential Processing. Written language was assessed through reading aloud, written comprehension, spontaneous writing, and copying and dictation tasks.

The word/pseudoword writing task (TEPPs)^38^ was used to assess written language skills. The participant was also asked to write down a series of words dictated by the examiner to exclude individuals with hearing impairment. Participants were allowed to complete the task using the hand with which they were most comfortable for writing. The percentage of correctly written Words (Regular, Irregular, Short, Long, Frequent, Infrequent) and Pseudowords (Short and Long), as well as a total score (72 stimuli), were calculated for the task. The influence of psycholinguistic variables on performance was assessed using the difference between the percentage of correctly written short and long words (length effect), regular and irregular words (regularity effect), frequent and infrequent words (frequency effect) and words and pseudowords (lexicality effects). Errors were also categorized as linguistic (verbal paragraphia, unfamiliarity with contextual rules, accentuation, regularization, lexicalization, neologisms, nonwords and non-answer) or peripheral (graphemic and graphomotor errors, rotated or mirrored writing, inclined or wavy writing, spacing between letters, tremor and perseveration).

## RESULTS

Different types of dysgraphia were classified based on comparisons between the performance of cases and controls (matched by gender, age and education). The following variables were used to categorize dysgraphia: number of errors, number of correct answers, psycholinguistic effects, types of error observed, as well as qualitative differences between the errors observed in patients and controls. Two patients were diagnosed as having lexical dysgraphia (LHD2 and RHD6), two as phonological dysgraphia (LHD3 and LHD5), one as mixed dysgraphia (LHD1) and one with peripheral dysgraphia (LHD4). These data are shown in Table 3.

**Table 3:** Types of dysgraphia according to percentage of correct answers, number of errors, psycholinguistic effects and types of errors in the TEPPs, and impairment in the Spontaneous Writing and Sentence Copying tasks of the NEUPSILIN-Af.

	Cases	Correct answers	Errors	Main psycholinguistic effects	Types of writing errors	Impairment in supplementary tasks
**Lexical** **dysgraphia**	LHD2	37	89	Regularity and frequency	Letter omission, graphomotor errors, regularization, graphemic paragraphia, spacing between letters	Spontaneous writing and sentence copying
	RHD6	49	42	Regularity and frequency	Regularization and graphemic paragraphia	Spontaneous writing
**Phonological dysgraphia**	LHD3	44	46	Word length, frequency and lexicality	Neologism, letter substitution, lexicalization, non-answer, semantic paragraphia	–
	LHD5	54	44	Word length and lexicality	Letter substitution and omission, neologisms, non-answers, lexicalization	Spontaneous writing and sentence copying
**Mixed or global dysgraphia**	LHD1	4	227	Regularity	Graphomotor, tremor, neologism, omission, perseveration, letter substitution, graphemic and verbal paragraphia	Spontaneous writing
**Peripheral dysgraphia**	LHD4	39	61	Word length and regularity	Mirrored writing, inclined writing, graphemic paragraphia, letter omission, addition and substitution	Spontaneous writing

LHD: left-hemisphere damage; RHD: right hemisphere damage.

## DISCUSSION

Patients LHD2 and RHD6 displayed regularity and frequency effects as well as regularization errors and graphemic paragraphias, suggesting the predominant use of phonological processing for writing words/pseudowords, and the presence of impairments to the lexical route (lexical dysgraphia).^4^ Most of the errors made by patient LHD2 consisted of letter omissions, which were observed in the spontaneous writing and sentence copying tasks of the NEUPSILIN-Af. Letter omissions often result from difficulties in the identification and production of words as a whole (lexical processing) and in phoneme-grapheme conversion when writing to dictation. Patient LHD2 also displayed letter formation errors (graphomotor) and excessive spacing between letters, neither of which were observed in case RHD6. Similar peripheral errors have been reported in cases of lexical dysgraphia, suggesting that damage to left cortico-subcortical circuits, which involve structures such as the putamen, the thalamus, and the premotor and sensorimotor cortices, can influence grapheme formation.^40^ Parieto-occipital lesions, such as those found in patient LHD2, have also been identified as an important cause of lexical dygraphia.^41^

Patient RHD6 had no symptoms of aphasia, suggesting the presence of right hemisphere language specialization, which is found in two percent of right-handed individuals.^42^ This patient's profile was similar to that reported by Rothi, Roeltgen and Kooistra (1987),^43^ who described the case of a right-handed adult with RHD which displayed both regularity effects and regularization errors. These authors suggested that patients with RHD may have difficulty using visual (or lexical) strategies to write words as a whole, relying instead on phonological strategies.

The patient with RHD assessed in the present study had a similar performance to that of an adult with posterior callosal damage described in a previous investigation, who was found to have difficulty writing Kanji (ideograms with no systematic relationship to corresponding spoken sounds).^44^ The study in question also found that the right hemisphere relies more on lexical-semantic processing than on phonological representations for word writing, possibly because phonological processes are more closely associated with the left hemisphere.^45^ Lexical processing strategies, on the other hand, tend to be more closely related to activation in frontal regions of the brain.^46^ Therefore, it is possible that the writing impairments observed in patient RHD6 as well as his lexical dysgraphia may have been caused by frontal damage to the right hemisphere. However, further studies of patients with frontal RHD are required to confirm this hypothesis. There is also a need for research involving larger samples of patients with RHD, since few studies have investigated the role of the right hemisphere in lexical processing, especially in Brazilian samples.

In the present study, two patients with LHD (LHD3 and LHD5) had significantly greater difficulty writing pseudowords as compared to real words (lexicality effect), and long words as compared to short ones (length effect). These error patterns are indicative of phonological dysgraphia. The patterns of brain damage observed in these patients corroborate the hypothesis that a complex neural network involving left perisylvian regions is responsible for phoneme-grapheme conversion in word and pseudoword writing, and that damage to this structure may be the cause of phonological dysgraphia.^2,18,19^

In addition to lexicality effects, these patients also exhibited neologisms, letter substitutions, lexicalization errors and non-answers in both the TEPPs and the Spontaneous Writing tasks of the NEUPSILIN-Af, probably due to impaired phoneme-grapheme conversion and to the exclusive reliance on lexical processing when writing words and pseudowords.^20^ These patients also made similar errors in spoken language tasks, in which phonological paraphasias, anomias and agrammatisms were observed. Some of the speech impairments displayed by patients with Broca's and Transcortical Motor Aphasia were also evident in their performance of word and pseudoword writing tasks.^47^

Semantic paragraphias, which are not commonly seen in phonological dysgraphia but are a common consequence of deep dysgraphia, were only observed in patient LHD3. During the writing-to-dictation task, the patient in question wrote down the word "birds" in response to the word "wing." According to Rapcsak et al. (2009),^19^ the degree of phonological processing deficits presented by patients can have a direct impact on the severity of their writing deficits. Given the presence of both semantic and orthographic errors in some of the most severely impaired participants, the authors proposed the existence of a continuum of written language impairment, comprising phonological dysgraphia on the least severe end of the spectrum, followed by deep dysgraphia and global or mixed dysgraphia, which are associated with similar severity levels. Therefore, it is possible that patient LHD3 may have transitioned from deep dysgraphia to the less severe phonological dysgraphia in the time since their stroke, either due to spontaneous language recovery, or to the beneficial effects of speech rehabilitation on phonological impairment. LHD3 is the youngest patient described in the present study with the longest time since stroke of 70 months, having already been through a long recovery period for their deficits. However, a longitudinal evaluation of this patient, involving pre- and post-rehabilitation assessments, would be required to confirm this hypothesis.

The performance of patient LHD1 was similar to that observed in patients with mixed or global aphasia, which lead to substantial impairments in word writing tasks due to its effects on both lexical and phonological processing.^20,21^ However, these patients had less difficulty writing regular words than irregular or pseudowords.

The most frequent errors in our sample were peripheral in nature. Tremor and graphomotor (poor letter formation) errors, for instance, were identified in all written stimuli. These errors are often observed in patients with basal ganglia lesions, which have a significant impact on motor control.^48^ Perseveration errors (repeated writing of previous stimuli) are also common in patients with damage to the basal ganglia. Patient LHD1 also exhibited both phonological and lexical errors, corroborating the idea that both types of processing are involved in word and pseudoword writing, although one may be more extensively involved than the other in certain cases.^15^

Luzzatti et al.^20^ suggested two main etiologies for mixed dysgraphia: auditory/phonological impairment (difficulty segmenting spoken words into sounds) or lexical-phonological output disturbances (grapheme selection in writing). The latter was more evident in LHD1, since the patient had adequate spoken language (including word repetition).

LHD1 developed expressive aphasia following her stroke, and had spontaneous speech recovery, never having received speech therapy. Therefore, it is possible that the stroke inflicted more damage to subcortical regions associated with word and pseudoword writing rather than to areas responsible for spoken language expression. These findings corroborated those of Scarone et al.,^48^ who demonstrated that the following cortical and subcortical regions were involved in word writing tasks: superior parietal cortex, supramarginal gyrus, second and third frontal gyri, supplemental motor area and insula. During the spontaneous recovery period, some patients may appear to recover from aphasia, but not from writing impairments, suggesting that these disturbances are caused by different lesions.^48^

LHD4 made predominantly mirrored and inclined writing errors, although graphemic errors were also observed (omission, addition and substitution of letters). The patient also displayed length and regularity effects, since fewer errors were observed in short and regular words. These features are characteristic of peripheral dysgraphia.^2,6^

Mirrored writing (writing some letters or the entire word in mirrored form) is a spatial error caused by impairments in the motor representation of letters, which is also observed in adults who are asked to write with their left hand.^49^ The motor sequences used for letter writing are associated with the right hands of right-handed individuals, so that a new motor program must be learned when individuals attempt to write with their left hands. Due to stroke-associated motor deficits, patient LHD4 performed the TEPPs with her non-dominant hand, which may explain the presence of mirrored writing in her responses to the task.

Patient LHD4 also had difficulty maintaining letter sequences while writing, possibly due to graphemic buffer damage.^2^ It is possible that these errors were caused by dysfunctions in working memory (namely, in the buffer component) during word writing.^23,24^ The graphemic buffer is also sensitive to word length effects, since longer words take up more of its capacity.^2,6^ Furthermore, patient LHD4 also displayed regularity effects, suggesting that the graphemic buffer may be more sensitive to certain letter sequences, such as those found in irregular words. This finding has been previously observed in a case of non-fluent aphasia by Gvion and Friedmann (2010).^50^

The errors exhibited by patient LHD4, which consisted mostly of the omission, addition and substitution of letters, are often observed in cases of graphemic buffer impairment. Graphemic paragraphias, consisting of phonologically plausible letter substitutions, were also observed. Although these are usually considered phonological errors, it is possible that in the case of this specific patient, they may have been caused by damage to the graphemic buffer. Similar errors have been reported in patients who suffered extensive LHD25, akin to that seen in patient LHD4.

In conclusion, the fact that dysgraphia was diagnosed in half the participants with LHD suggests that this hemisphere plays an important role in word writing. The presence of lexical dysgraphia in a patient with RHD also underscores the need for further studies of the role of the right hemisphere in word processing.

The fact that two patients with LHD displayed poor performance and made several errors in the TEPPs, in spite of an absence of aphasia, suggested that the cognitive mechanisms involved in spoken language are distinct from those responsible for writing. On the other hand, patients with aphasia made similar errors on both spoken and written tasks, suggesting that, in more severe cases, both spoken and written language may be impaired, corroborating the hypothesis of a continuum of severity in dysgraphia. Results such as those of the present study help advance knowledge on written word processing, and may serve as a basis for neuropsychological interventions which focus specifically on the different patterns of impairment observed in each type of dysgraphia.

## References

[1] Ardila A, Rosselli M, Ardila A, Rosselli M (2007). Agrafia. Neuropsicología Clínica.

[2] Rapcsak S, Beeson PM (2002). Agraphia. Enciclopedia of the Human Brain.

[3] Henry ML, Beeson PM, Stark AJ, Rapcsak SZ (2007). The role of left perisylvian cortical regions in
spelling. Brain Lang.

[4] Rapcsak SZ, Beeson PM (2004). The role of left posterior inferior temporal cortex in
spelling. Neurology.

[5] Rapcsak SZ, Rubens AB (1990). Disruption of semantic influence on writing following a left
prefrontal lesion. Brain Lang.

[6] Carthery MT, Parente MAMP, Ortiz KZ (2010). Agrafias adquiridas - Introdução histórica e
classificação. Distúrbios Neurológicos Adquiridos.

[7] Ardila A, Rosselli M (1993). Spatial agraphia. Brain Cogn.

[8] Cubelli R, Guiducci A, Consolmagno P (2000). Afferent dysgraphia after right cerebral stroke: An autonomous
syndrome?. Brain Cogn.

[9] Seki K, Ishiai S, Koyama Y (1998). Effects of unilateral spatial neglect on spatial agraphia of Kana
and Kanji letters. Brain Lang.

[10] Beeson PM, Rapcsak SZ, Plante E (2003). The neural substrates of writing: A functional magnetic resonance
imaging study. Aphasiology.

[11] Coltheart M (2006). Acquired dyslexias and the computational modeling of
reading. Cogn Neuropsychol.

[12] Houghton G, Zorzi M (2003). Normal and impaired spelling in a connectionist dual-route
architecture. Cogn Neuropsychol.

[13] Plaut DC, McClelland JL, Seidenberg MS, Patterson K (2006). Understanding normal and impaired word reading: Computational
principles in quasiregular domains. Psychol Rev.

[14] Rapcsak SZ, Henry ML, Teague SL, Carnahan SD, Beeson PM (2007). Do dual-route models accurately predict reading and spelling
performance in individuals with acquired alexia and
agraphia?. Neuropsychologia.

[15] Ellis AW, Batista Dayse (1995). Leitura, escrita, dislexia. Uma análise cognitiva.

[16] Lecours AR, Parente MAMP (1997). Dislexia: Implicações do sistema de escrita do
português.

[17] Coltheart M, Rastle K, Perry C, Langdon R, Ziegler T (2001). DRC: Dual-route cascaded model of visual word recognition and
reading aloud. Psychol Rev.

[18] Henry ML, Beeson PM, Stark AJ, Rapcsak SZ (2007). The role of left perisylvian cortical regions in
spelling. Brain Lang.

[19] Rapcsak SZ, Beeson PM, Henry ML (2009). Phonological dyslexia and dysgraphia: Cognitive mechanisms and
neural substrates. Cortex.

[20] Luzzatti C, Laiacona M, Allamano N, De Tanti A, Inzaghi MG (1998). Writing disorders in Italian aphasic patients: A multiple
single-case study of dysgraphia in a language with shallow
orthography. Brain.

[21] Laiacona M, Capitani E, Zonca G, Scola I, Saletta P, Luzzatti C (2009). Integration of lexical and sublexical processing in the spelling
of regular words: A multiple single-case study in Italian dysgraphic
patients. Cortex.

[22] Jefferies E, Sage K, Ralph MAL (2007). Do deep dyslexia, dysphasia and dysgraphia share a common
phonological impairment?. Neuropsychologia.

[23] Caramazza A, Miceli G, Villa G (1986). The role of the (output) phonological buffer in reading, writing
and repetition. Cogn Neuropsychol.

[24] Miceli G, Capasso R (2006). Spelling and dysgraphia. Cognitive Neuropsychol.

[25] Miceli G, Benvegnù B, Capasso R, Caramazza A (1997). The independence of phonological and orthographic lexical forms:
Evidence from aphasia. Cognitive Neuropsychol.

[26] Di Pietro M, Schnider A, Ptak R (2011). Peripheral dysgraphia characterized by the co-occurrence of case
substitutions in uppercase and letter substitutions in lowercase
writing. Cortex.

[27] Rapp B, Caramazza A (1997). From graphemes to abstract letter shapes: Levels of
representation in written spelling. J Exp Psychol Hum Percept Perform.

[28] Sakurai Y, Onuma Y, Nakazawa G (2007). Parietal dysgraphia: Characterization of abnormal writing stroke
sequences, character formation and character recall. Behav Neurol.

[29] Goodglass H, Kaplan E, Barresi B (2001). Boston Diagnostic Aphasia Examination Short Form.

[30] Radanovic M, Mansur LL, Azambuja MJ, Porto CS, Scaff M (2004). Contribution to the evaluation of language disturbances in
subcortical lesions: A pilot study. Arq Neuropsiquiatr.

[31] Pawlowski J, Remor E, Parente MAMP, Salles JF, Fonseca RP, Bandeira DR (2012). The inﬂuence of reading and writing habits associated with
education on the neuropsychological performance of Brazilian
adults. Read Writ.

[32] Yesavage JA, Brink TL, Rose TL, Lurn O (1983). Development and validation of a geriatric depression screening
scale: a preliminary report. J Psychiatr Res.

[33] Gorenstein C, Pang WY, Argimon IL, Werlang BSG (2011). BDI-II - Inventário de depressão de Beck.

[34] Chaves ML, Izquierdo I (1992). Differential diagnosis between dementia and depression: A study
of efficiency increment. Acta Neurol Scand.

[35] Moreira L, Schlottfeldt CG, Paula JJ (2011). Estudo Normativo do Token Test versão reduzida: Dados
preliminares para uma população de idosos
brasileiros. Rev Psiquiatr Clín.

[36] Fontoura DR, Rodrigues JC, Parente MAMP, Fonseca RP, Salles JF (2011). Adaptação do Instrumento de Avaliação
Neuropsicológica Breve NE-UPSILIN para avaliar pacientes com afasia
expressiva: NEUPSILIN-Af. Ciênc Cogn.

[37] Fontoura DR, Rodrigues JC, Mansur L, Monção AM, Salles JF (2013). Neuropsycholinguistic profile of patients post-stroke in the left
hemisphere with expressive aphasia. Rev Neuropsic Neuropsiq Neurocien.

[38] Rodrigues JC, Salles JF (2013). Tarefa de escrita de palavras/pseudopalavras para adultos:
abordagem da neuropsicologia cognitiva. Letras de Hoje.

[39] Schwartz MF, Dell GS (2010). Case series investigations in cognitive
neuropsychology. Cogn Neuropsychol.

[40] Sakurai Y, Yoshida Y, Sato K, Sugimoto I, Mannen T (2011). Isolated thalamic agraphia with impaired grapheme formation and
micrographia. J Neurol.

[41] Roeltgen DP, Heilman KM (1984). Lexical agraphia: Further support for the two-strategy hypothesis
of linguistic agraphia. Brain.

[42] Springer JA, Binder JR, Hammeke TA (1999). Language dominance in neurologically normal and epilepsy
subjects: A functional MRI study. Brain.

[43] Rothi LJG, Roeltgen DP, Kooistra CA (1987). Isolated lexical agraphia in a righthanded patient with a
posterior lesion of the right cerebral hemisphere. Brain Lang.

[44] Yamadori A, Nagashima T, Tamaki N (1983). Ideogram writing in a disconnection syndrome. Brain Lang.

[45] Rapcsak SZ, Beeson PM, Rubens AB (1991). Writing with the right hemisphere. Brain Lang.

[46] Hayashi A, Nomura H, Mochizuki R (2011). Neural substrates for writing impairments in Japanese patients
with mild Alzheimer's disease: A SPECT study. Neuropsychologia.

[47] Helm-Estabrooks N, Albert ML (2003). Manual of aphasia and aphasia therapy.

[48] Scarone P, Gatignol P, Guillaume S, Denvil D, Capelle L, Duffau H (2009). Agraphia after awake surgery for brain tumor: New insights into
the anatomo-functional network of writing. Surg Neurol.

[49] Balfour S, Borthwick S, Cubelli R, Della Sala S (2007). Mirror writing and reversing single letters in stroke patients
and normal elderly. J Neurol.

[50] Gvion A, Friedmann N (2010). Letter position dysgraphia. Córtex.

